# Tau aggregation following subcortical hemorrhage

**DOI:** 10.1007/s00259-024-06662-5

**Published:** 2024-03-04

**Authors:** Elena Jaeger, Gérard N. Bischof, Oezguer A. Onur, Marc Schlamann, Alexander Drzezga

**Affiliations:** 1 Department of Nuclear Medicine, University of Cologne, University Hospital of Cologne, Kerpenerstraße 62, 50931 Cologne, Germany; 2 Department of Neurology, University of Cologne, University Hospital of Cologne, Cologne, Germany; 3 Department of Diagnostic and Interventional Radiology, University of Cologne, University Hospital of Cologne, Cologne, Germany; 4 Research Center Juelich, Institute for Neuroscience and Medicine II, Molecular Organization of the Brain, Juelich, Germany; 5 Research Center Juelich, Institute for Neuroscience and Medicine III, Cognitive Neuroscience, Juelich, Germany; 6 German Center for Neurodegenerative Diseases (DZNE), Bonn-Cologne, Germany

A 54-year-old male presented in the department of neurology with cognitive-amnestic deficits and episodes of dystonic spasms in the left hand. In 2020, the patient had a right basal ganglia hemorrhage accompanying a paresis of the left hand. Due to the paresis, he was not able to define the exact symptom onset of the dystonic spasms. Cerebrospinal fluid (CSF) diagnostics (05/2023) showed an increase in total tau as well as phospho-tau. Amyloid-ß-1-42 was within normal range. The patient was suspected to suffer from a corticobasal syndrome and a tau-PET/CT scan was performed.

Tau-PET/CT imaging ([^18^F]PI-2620, 0–75 min. p. i.) revealed a higher radiotracer-uptake mainly in the right thalamus extending towards the right striatum/globus pallidus compared to the left side. No further tau-retention was detected. High spatial correspondence between the location of the intracerebral hemorrhage and the increased [^18^F]PI-2620-PET uptake was observed.

Tau-PET is employed for the detection of neurodegenerative tauopathies, particularly Alzheimer’s disease (AD). In AD, predominantly cortical distribution patterns are observed [1]. For second-generation tau-PET tracers like [^18^F]PI-2620, potential diagnostic value to detect non-AD tauopathies such as progressive supranuclear palsy (PSP) or corticobasal degeneration (CBD) has been discussed [2]. In these disorders, basal ganglia uptake of the tracer is expected. However, post-mortem analyses have shown that tau-deposition can also occur after ischemic or hemorrhagic events [3]. This suggests that the tracer uptake observed in the current case, although specifically indicating tau-pathology may be the consequence of tissue damage following the intracerebral hemorrhage rather than of a neurodegenerative disease.

**Figure:**
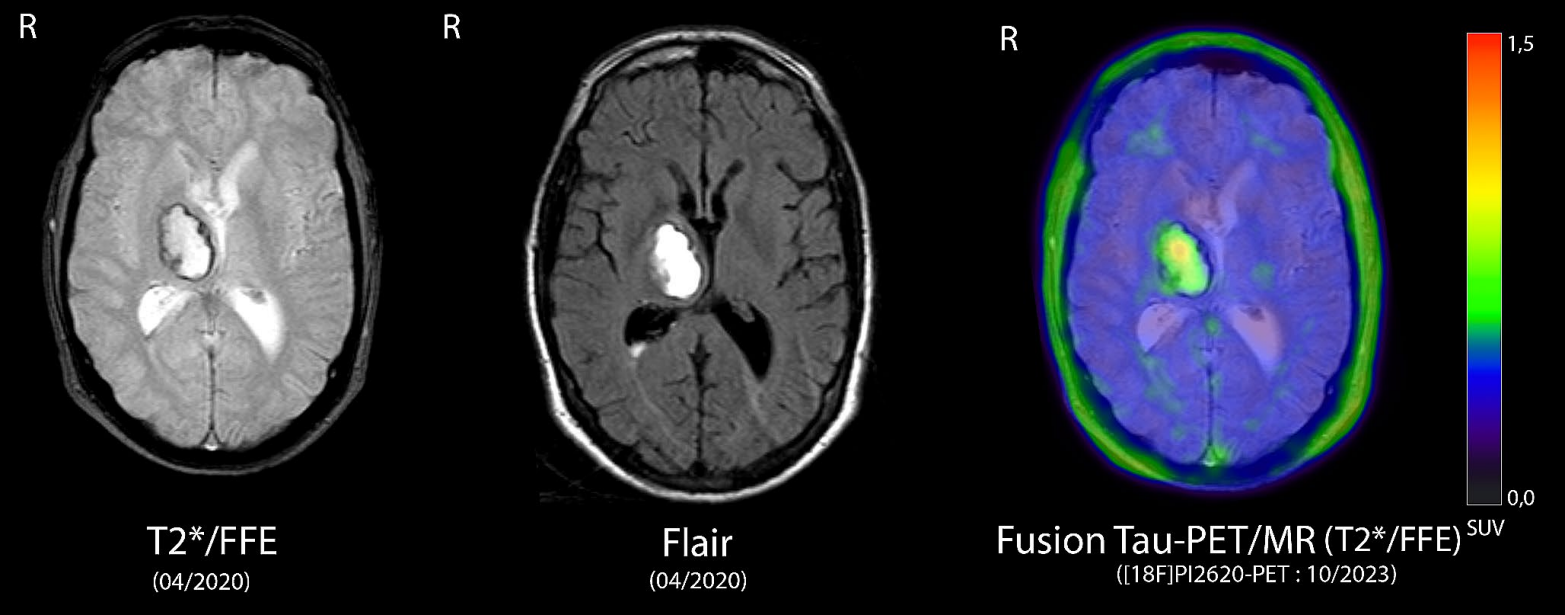

## References

[1] Dronse J, Fliessbach K, Bischof GN, von Reutern B, Faber J, Hammes J (2017). In vivo patterns of Tau Pathology, Amyloid-β Burden, and neuronal dysfunction in clinical variants of Alzheimer’s Disease. J Alzheimers Dis.

[2] Katzdobler S, Nitschmann A, Barthel H, Bischof G, Beyer L, Marek K (2023). Additive value of [18F]PI-2620 perfusion imaging in progressive supranuclear palsy and corticobasal syndrome. Eur J Nucl Med Mol Imaging.

[3] Hatsuta H, Takao M, Nogami A, Uchino A, Sumikura H, Takata T (2019). Tau and TDP-43 accumulation of the basal nucleus of meynert in individuals with cerebral lobar infarcts or hemorrhage. Acta Neuropathol Commun.

